# Supplementary material for: Population genomic signatures of the oriental fruit moth related to the Pleistocene climates
Source: Commun Biol. 2022 Feb 17;5:142. doi: 10.1038/s42003-022-03097-2 (PMC8854661; doi:10.1038/s42003-022-03097-2)
Supplement: Supplementary file 2 — Supplementary Information [file 42003_2022_3097_MOESM2_ESM.pdf]

Supplementary information

Population genomic signatures of the oriental fruit moth related to the Pleistocene climates

Li-Jun Cao [1, #], Wei Song [1,2, #], Jin-Cui Chen [1], Xu-Lei Fan [1], Ary Anthony Hoffmann [3], Shu-Jun Wei [1, *]

1. Institute of Plant and Environmental Protection, Beijing Academy of Agriculture and Forestry Sciences, 9 Shuguanghuayuan Middle Road, Haidian District, Beijing 100097, China
2. Beijing Key Laboratory for Forest Pests Control, Beijing Forestry University, Beijing, 100083, China
3. School of BioSciences, Bio21 Institute, University of Melbourne, Parkville, Victoria, Australia

# these authors have equal contributions.

*** Corresponding author**:

Shu-Jun Wei, Institute of Plant and Environmental Protection, Beijing Academy of Agriculture and Forestry Sciences, 9 Shuguanghuayuan Middle Road, Haidian District, Beijing 100097, China; Tel: +86 10 51503439; E-mail: shujun268@163.com

Supplementary Table 1: Summary statistics of generated sequencing data for *Grapholita molesta* genome assembly and annotation in this study

Library code	Library type	Sequencing instrument	Size (bp)	Coverage
GM_PE	DNA Pair-End (PE) library	Illumina HiSeq X10	48,722,298,600	94.11
GM_ONT	DNA Oxford NanoPore library	Oxford NanoPore	53,706,537,032	103.74
GM_HiC	DNA Hi-C library	Illumina HiSeq X10	120,847,620,900	233.43
GM_Egg	RNA-Seq library	Illumina NovaSeq	4,843,131,000	\
GM_Larve	RNA-Seq library	Illumina NovaSeq	4,345,527,750	\
GM_Pupa	RNA-Seq library	Illumina NovaSeq	4,517,857,500	\
GM_Adult	RNA-Seq library	Illumina NovaSeq	5,817,045,150	\

Genomic DNA was extracted using the DNeasy tissue kit (Qiagen, Hilden, Germany) for Illumina library construction and using the MagAttract HMW DNA kit (Qiagen, Hilden, Germany) for NanoPore library construction; the Hi-C proximity ligation library was constructed following the method of Berkum, et al. [1]. Briefly, the genome was digested by the restriction enzyme *MboI*. Fragments were sheared into 200-600 bp. A-tailing by Klenow (exo-) and then Illumina paired-end sequencing adapters were added to the fragmented ends. The libraries were amplified by 12 PCR cycles and sequenced on the Illumina HiSeq X Ten platform; RNA-seq libraries were prepared using VAHTSTM mRNA-seq V2 Library Prep Kit (Vazyme, Nanjing, China) and sequenced on the Illumina NovaSeq platform.

Supplementary Table 2: Summary of non-coding RNAs in the genome of *Grapholita molesta*

Class	Type	Number
rRNA count	8s_rRNA	61
	5s_rRNA	63
tRNA Count	tRNAs decoding Standard 20 AA	8853
	Selenocysteine tRNAs (TCA)	14
	Possible suppressor tRNAs (CTA, TTA)	13
	tRNAs with undetermined/unknown isotypes	163
	Predicted pseudogenes	20743
Total tRNAs		29786
tRNAs with intron		203

Supplementary Table 3: Summary of repeat elements in the genome of *Grapholita molesta*

Items	No. elements	Length (bp)	Percentage of sequence (%)
**Retroelements**	231642	41362559	7.99
SINEs	26922	4947739	0.96
LINEs	200582	33924345	6.55
LTR elements	4138	2490475	0.48
**DNA transposons**	8670	1730875	0.33
Unclassified	25907	3076572	0.59
Total interspersed repeats		46170006	8.92
**Small RNA**	19652	4014531	0.78
**Satellites**	2	267	0
**Simple repeats**	135315	5832340	1.13
**Low complexity**	19218	906543	0.18

Retrotransposable elements, known to be the dominant form of repeats, constituted a large part of the genome and included the most abundant subtypes, such as long terminal repeat elements (LTRs), long interspersed nuclear elements (LINEs) and short interspersed nuclear elements (SINEs). We predicted 41,362,559 bp retroelements (including 4,947,739 bp SINEs, 33,924,345 bp LINEs and 2,490,475 bp LTRs) in the OFM genome based on Insecta RepBase libraries, which grouped into 231642 elements and occupied 7.99% of the assembly. We annotated 8670 DNA transposons (1,730,875 bp, 0.33 %), 19,652 small RNA (4014531 bp, 0.78 %) and 135317 Tandem Repeats (TRs) (2 satellites and 135,315 simple repeats). We identified 19218 low complexity repeat elements occupying 0.18 % of the whole genome.

Supplementary Table 4: Number of genes in five detoxification families across 12 genomes from the Lepidoptera

Species	P450	GST	CCE	ABC	UGT	GR	IR	OBP	OR	HSP	Reference
Pxyl	163	55	85	219	38	68	61	91	225	56	You, et al. [2]
Csas	95	76	63	93	27	41	49	56	176	54	Cao, et al. [3]
Gmol	186	56	119	128	47	95	81	64	295	46	This study
Cpom	136*	30*	73*	47*	30*	65*	39*	50*	85*	43	Wan, et al. [4]
Dple	107	35	73	76	47	74	62	68	253	63	Zhan, et al. [5]
Tni	143	51	122	71	68	85	74	91	274	55	Chen, et al. [6]
Slit	182	66	153	223	64	107	62	87	261	58	Cheng, et al. [7]
Harm	122	57	105	76	54	120	71	79	257	53	Song, et al. [8]
Bman	94	37	94	64	48	69	56	74	218	59	Xiang, et al. [9]
Bmor	156	51	149	108	50	85	57	93	243	56	Xia, et al. [10]
Msex	164	66	137	103	51	89	76	79	261	67	Kanost, et al. [11]
Gmel	137	44	75	72	58	95	81	64	295	55	Lange, et al. [12]
Ofur	126	48	115	112	46	93	67	75	270	59	Ma, et al. [13]

*, data from Wan, et al. [4]; the other data were manually identified in our study. Pxyl, *Plutella xylostella*; Gmol, *Grapholita molesta*; Cpom, *Cydia pomonella*; Dple, *Danaus plexippus*; Tni, *Trichoplusia ni*; Slit, *Spodoptera litura*; Harm, *Helicoverpa armigera*; Bman, *Bombyx mandarina*; Bmor, *Bombyx mori*; Msex, *Manduca sexta*; Gmel, *Galleria mellonella*; Ofur, *Ostrinia furnacalis*.

Methods of gene family annotation

Protein-coding genes from available genomes of two Coleoptera, two Diptera and another 11 Lepidoptera were retrieved from the NCBI genome database for comparative analysis. Orthologs were identified using OrthoFinder v2.2.7 [14] under default parameters. MAFFT v7.450 [15] was used to align amino acid sequences of 1:1:1 orthologous gene with the G-INS-I algorithm. The phylogenetic tree was inferred using an approximately-maximum-likelihood method implemented in FastTree v2.1.10 [16]. We used r8s [17] to estimate the divergent times among species with divergence times of two nodes, i.e., *Tribolium castaneum* and *Anoplophora glabripennis* [18], *Trichoplusia ni* and *S. litura* as calibrations. The Computational Analysis module of gene Family Evolution (CAFE) version 3.1 [19] was used to analyze gene family expansion and contraction.

To explore possible genomic components related to environmental adaption in the OFM and the other tortricid moth, *C. pomonella*, we manually annotated detoxification genes, chemosensory genes, and heat shock proteins (HSP) genes and compared these gene families among 13 representative genomes of Lepidoptera. The detoxification genes include five families of cytochrome P450 monooxygenases (P450s), glutathione-s transferases (GSTs), ATP-binding cassette transporters (ABCs), UDP-glycosyltransferases (UGTs), and carboxyl/cholinesterases (CCEs). The chemosensory genes include four families of olfactory receptors (ORs), gustatory receptors (GRs), Ionotropic receptors (IRs), and odorant-binding proteins (OBPs). We used both model-based and similarity-based methods to annotate these gene families. For model-based identification, the Hidden Markov models (HMMs) were downloaded from Pfam 32.0 database (September 2018; [20]) and run with HMMER v3.3 [21]. The corresponding HMM model not found in the Pfam database was manually trained using HMMER under the default parameters. For similarity-based identification, we used orthologs from *D. melanogaster*, *B. mori*, *Aedes aegypti*, *Anopheles gambiae,* and *C. pomonella* to search against target genomes using BLAST v2.2.31 [22] with an e-value cutoff of $1e^{-5}$. An automatic BITACORA v1.0 [23] pipeline (full mode) was used to conduct the HMMER and BLAST analyses. The annotated genes were filtered manually based on gene length and the presence of conserved domains by removing genes shorter than 80 amino acids and those lacking conserved domains.

Discussion on the adaptation of OFM from the aspect of gene family

In insects, expansion or contraction of gene families provides a signature of evolution that may be linked to changes in species which are linked to traits such as invasiveness, metabolic plasticity, feeding habits, pesticides resistance, migration, and symbiotic relationships with a microorganism [24]. We focused on gene families of detoxification genes, chemosensory genes, and heat shock proteins (HSP), which may be associated with environmental adaptation and dispersal. There is a contraction of HSP gene family, which has been linked to stressful conditions as well as development in insects, in OFM and another tortricid moth, *C. pomonella*. Perhaps the low numbers of these genes relate to the lack of thermal stress experienced by these species, given their distribution in temperate and tropical high upland regions [25], and a larval habitat which involves larvae being located in protected environments like fruit or buds which can mitigate the effect of environmental

temperature extremes.

An expansion of P450 gene families has been detected in many invasive species, such as *Culex quinquefasciatus*, *P. xylostella, Ceratitis capitata*, *Bemisia tabaci*, *Blattella germanica,* and *C. pomonella*, most of which show resistance to many chemical pesticides [2,26-29]. In both the tortricid moths, the high number of P450 genes may indicate enhanced abilities to cope with phytochemical or synthetic toxins (Wan *et al*. 2019), even though pesticide resistance is not common in the OFM (but see Jones, et al. [30] and Kanga, et al. [31]). It would be interesting to examine signatures of selection in these genes in regions away from refugial areas where pesticides are regularly applied.

A noteworthy feature of the OFM genome is the high number of IRs and ORs compared to other investigated Lepidoptera genomes, including the codling moth *C. pomonella.* A previous study has shown that a duplication event of an OR gene facilitated both host plants and mates (Wan et al. 2019). The higher number of detoxification and receptor genes in the OFM genome than *C. pomonella* may relate to its wider host range, given that *C. pomonella* only feeds on fruit, while the OFM also feeds on twigs of peaches, especially in early spring. These genome characteristics may also lead to higher invasiveness of the OFM compared to other tortricid moths.

Supplementary Table 5: Sampling information for 15 populations of oriental fruit moth (OFM)*Grapholita molesta* for genotyping. See Fig. 2 for the location of the populations.

Population code	Collection location	Long.	Lat.	Collection date	Number	Genotyping method
YNHH	Honghe, Yunnan	103.52	23.34	Jul, 2016	11	Re-sequencing
SCCD	Chengdu, Sichuan	104.31	30.54	Jul, 2016	11	Re-sequencing
BJPG	Pinggu, Beijing City	117.12	40.14	Jun, 2010	9	Re-sequencing
LNSY	Liaoning Province, Shenyang	123.43	41.81	Sep, 2010	25	KASP
HBSJ	Hebei Province, Shijiazhuang	114.51	38.04	May, 2010	24	KASP
SDQD	Shandong Province, Qingdao	120.43	36.16	Jun, 2010	24	KASP
SXYA	Shanxi Province, Yanan	109.49	36.59	Aug, 2010	24	KASP
FJND	Fujian Province, Ningde	119.55	26.67	Jul, 2011	24	KASP
GZGY	Guizhou Province, Guiyang	106.67	26.34	Aug, 2016	11	KASP
SCGY	Sichuan Province, Guangyuan	105.89	32.64	Jul, 2016	14	KASP
SCBZ	Sichuan Province, Bazhong	106.66	31.84	Jul, 2016	9	KASP
SCNC	Sichuan Province, Nanchong	105.98	31.16	Jul, 2016	24	KASP
YNQJ	Yunnan Province, Qujing	104.15	26.28	May, 2016	9	KASP
YNKM	Yunnan Province, Kunming	102.67	25.16	Apr, 2016	26	KASP
YNBS	Yunnan Province, Baoshan	99.17	25.15	Jul, 2016	10	KASP

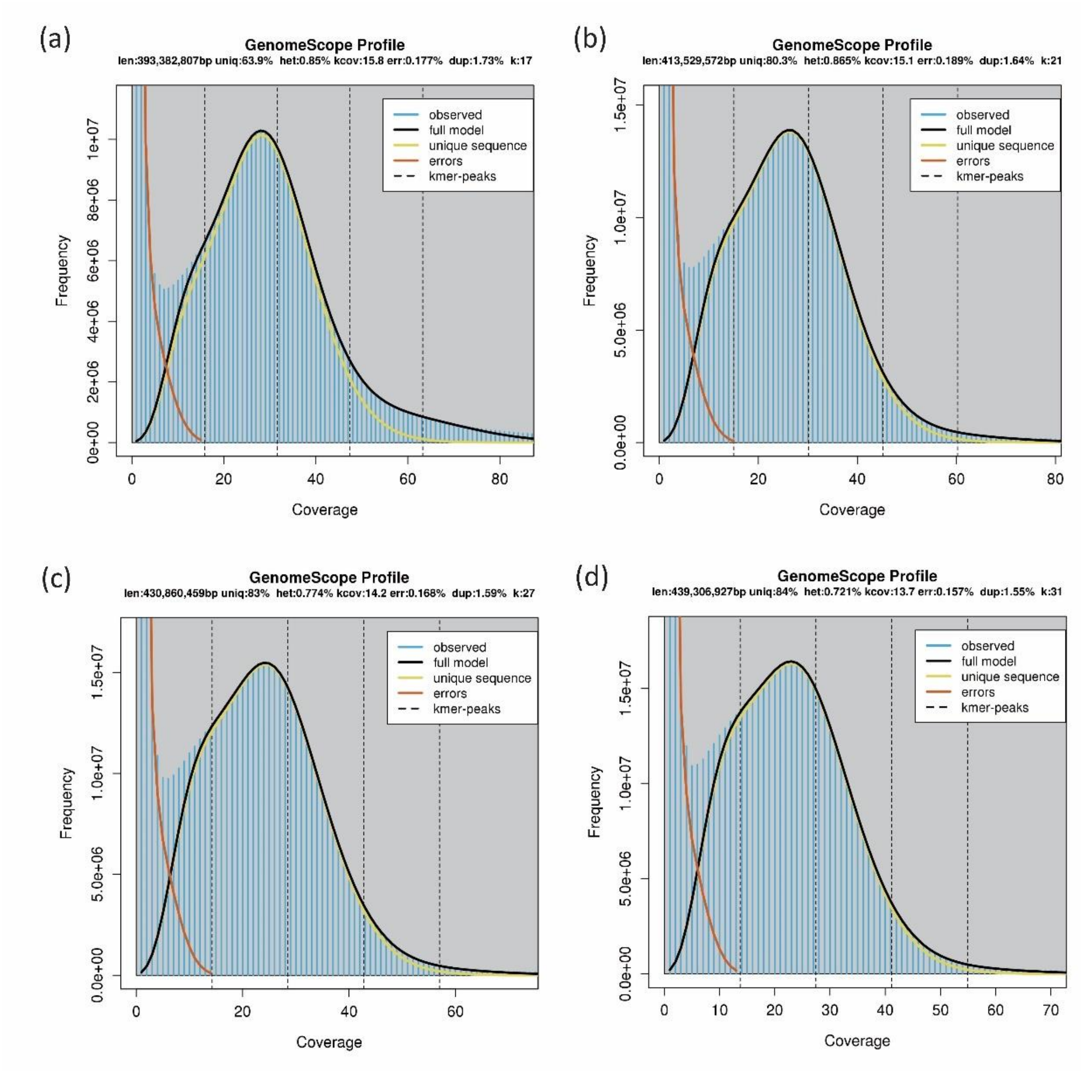

Supplementary Fig. 1: Genomescope estimation of (Busck)genome size, heterozygosity and rate of duplication for the oriental fruit moth *Grapholita molesta* when k-mer= 17 (a), 21 (b),27 (c)and 31 (d).

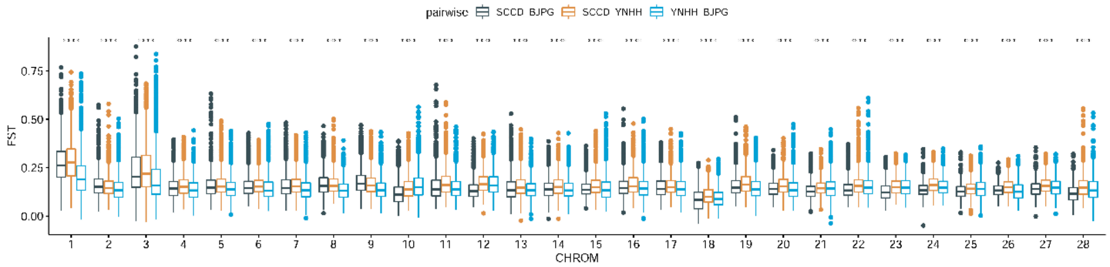

Supplementary Fig. 2: Boxplot of pairwise population F_{ST} across different chromosomes. The average F_{ST} values of the neo-Z chromosome (chr1)was the highest, followed by chr3. SCCD population showed a relatively high differentiation from other two populations across most of chromosomes, especially on neo-Z chromosome. All bars denote the largest value within 1.5 times interquartile range above 75th percentile and the smallest value within 1.5 times interquartile range below 25th percentile.

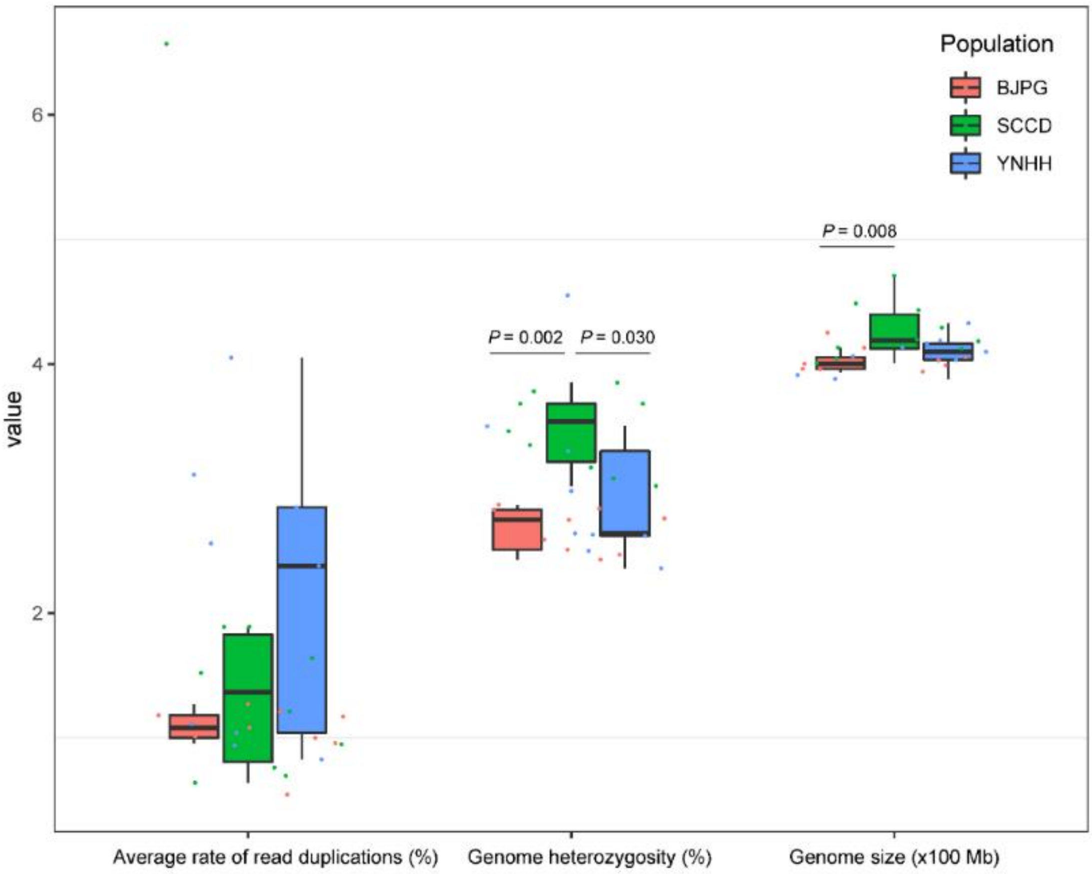

Supplementary Fig. 3: Box plots for estimated genome features of the OFM for sequenced individuals. Data are presented by population. The k-mers were counted by jellyfish v 2.2.10 with a 17-base oligonucleotide. Genome size, heterozygosity and rate of duplication were estimated using GenomeScope v1.0. Population codes are from Table S5. Nine individuals from BJPG, 11 individuals from SCCD, and 11 individuals from YNHH were used. P values are from Wilcoxon-test. All bars denote the largest value within 1.5 times interquartile range above 75th percentile and the smallest value within 1.5 times interquartile range below 25th percentile.

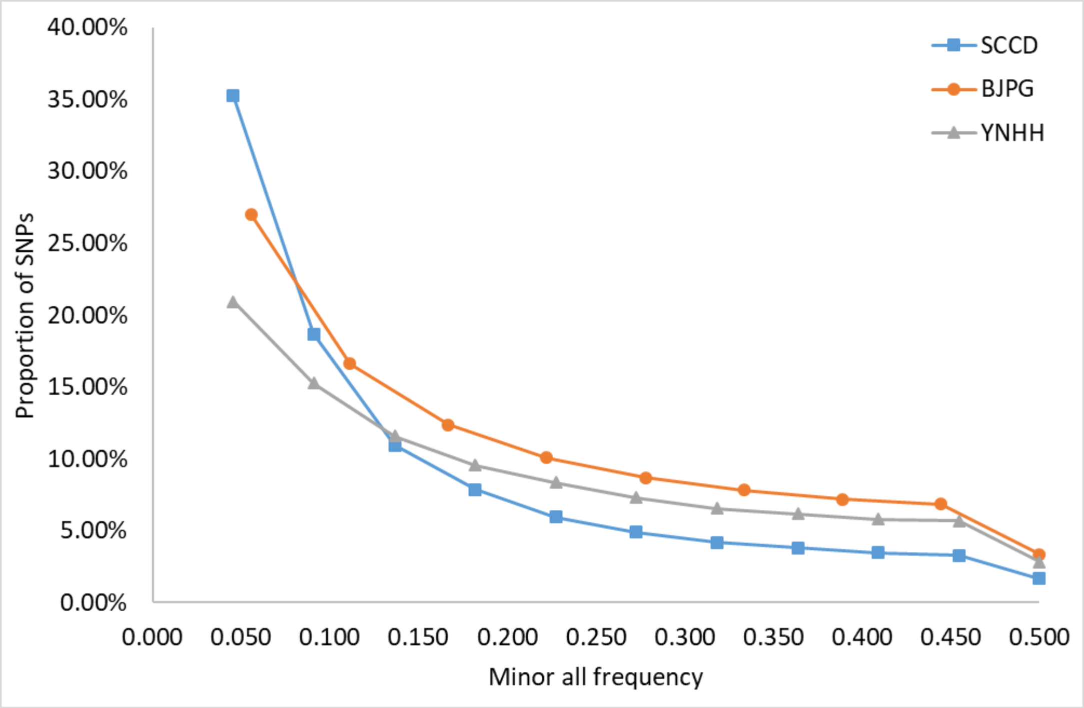

Supplementary Fig. 4: SNP allele frequency spectrum distribution for oriental fruit moth *Grapholita molesta* in the three populations. The x-axis represents minor allele frequency with the corresponding proportion of SNPs on the y-axis.

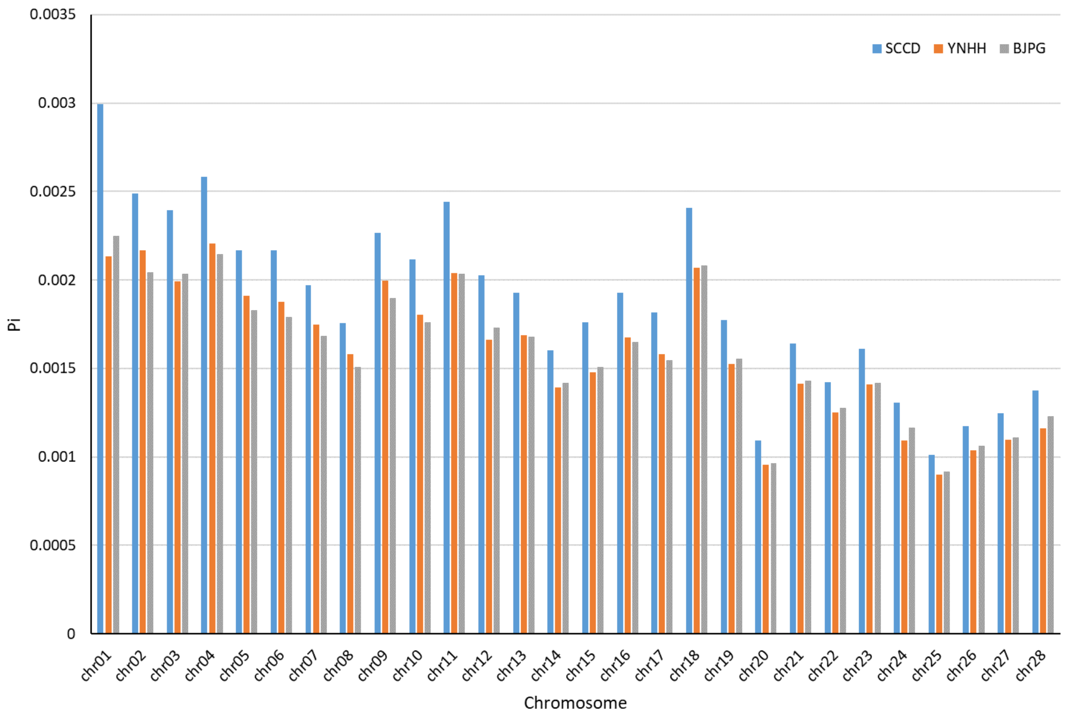

Supplementary Fig. 5: Nucleotide diversity (π)of the three OFM populations presented separately for each chromosome.

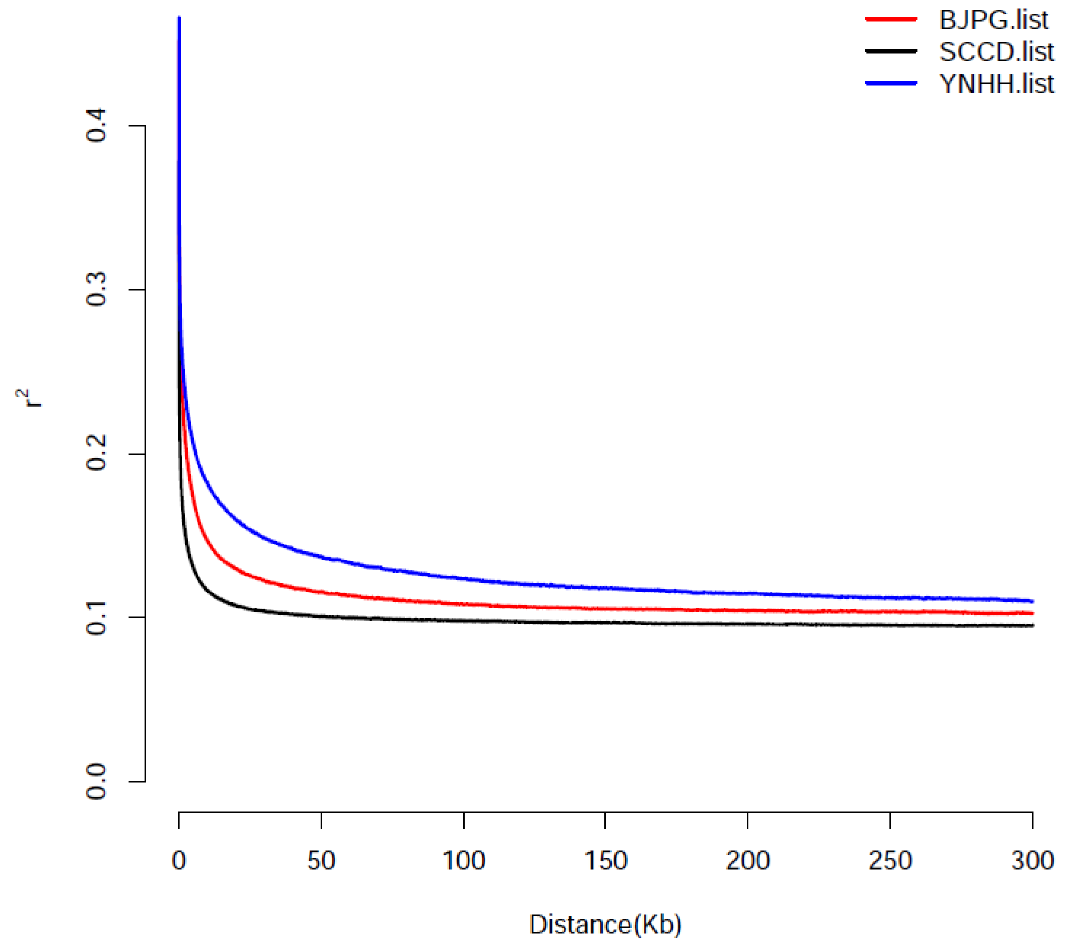

Supplementary Fig. 6: Average linkage disequilibrium between pairs of SNPs in each population of OFM. Four populations of PFM and two populations of OFM show particularly high levels of LD relative to the other populations, as well as lower genetic diversity, which might be caused by strong bottlenecks or biased sampling.

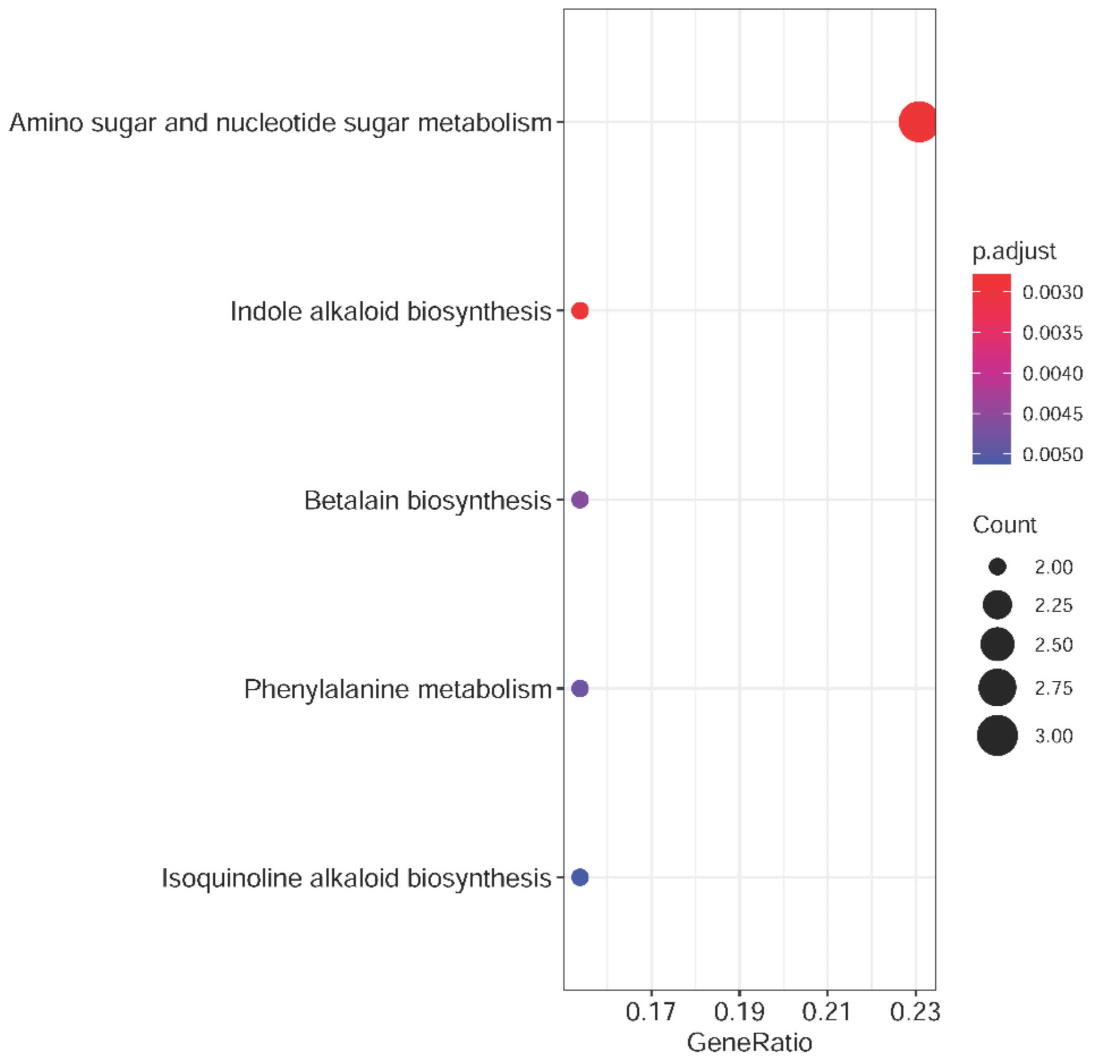

Supplementary Fig. 7: Genes and Genomes (KEGG)enrichment analysis of genes involved northward dispersal of OFM. The left column contains KEGG pathway terms. The dot size stands for the different numbers of DEGs falling within the category. The color gradient from red to blue indicates the range of p values from 0.0028 to 0.0051.

Supplementary References

1 Berkum, N. L. V., Lieberman-Aiden, E., Williams, L., Imakaev, M. & Lander, E. S. Hi-C: A method to study the three-dimensional architecture of genomes. *Journal of Visualized Experiments Jove* **39**, 1-7 (2010).

2 You, M. S. *et al.* A heterozygous moth genome provides insights into herbivory and detoxification. *Nature Genetics* **45**, 220-225 (2013).

3 Cao, L. J. *et al.* Chromosome-level genome of the peach fruit moth *Carposina sasakii* (Lepidoptera: Carposinidae) provides a resource for evolutionary studies on moths. *Mol Ecol Resour* **21**, 834-848, doi:10.1111/1755-0998.13288 (2021).

4 Wan, F. H. *et al.* A chromosome-level genome assembly of *Cydia pomonella* provides insights into chemical ecology and insecticide resistance. *Nat Commun* **10**, doi.org/10.1038/s41467-41019-12175-41469, doi:ARTN 4237
10.1038/s41467-019-12175-9 (2019).

5 Zhan, S., Merlin, C., Boore, J. L. & Reppert, S. M. The monarch butterfly genome yields insights into long-distance migration. *Cell* **147**, 1171-1185, doi:10.1016/j.cell.2011.09.052 (2011).
6 Chen, W. *et al.* A high-quality chromosome-level genome assembly of a generalist herbivore, *Trichoplusia ni*. *Molecular Ecology Resources* **19**, 485-496, doi:10.1111/1755-0998.12966 (2019).
7 Cheng, T. *et al.* Genomic adaptation to polyphagy and insecticides in a major East Asian noctuid pest. *Nature Ecology & Evolution* **1**, 1747-1756, doi:10.1038/s41559-017-0314-4 (2017).
8 Song, S. V., Downes, S., Parker, T., Oakeshott, J. G. & Robin, C. High nucleotide diversity and limited linkage disequilibrium in *Helicoverpa armigera* facilitates the detection of a selective sweep. *Heredity (Edinb)* **115**, 460-470, doi:10.1038/hdy.2015.53 (2015).
9 Xiang, H. *et al.* The evolutionary road from wild moth to domestic silkworm. *Nature Ecology & Evolution* **2**, 1268-1279, doi:10.1038/s41559-018-0593-4 (2018).
10 Xia, Q. *et al.* A draft sequence for the genome of the domesticated silkworm (*Bombyx* mori). *Science* **306**, 1937-1940, doi:10.1126/science.1102210 (2004).
11 Kanost, M. R. *et al.* Multifaceted biological insights from a draft genome sequence of the tobacco hornworm moth, Manduca sexta. *Insect Biochemistry and Molecular Biology* **76**, 118-147, doi:10.1016/j.ibmb.2016.07.005 (2016).
12 Lange, A. *et al.* Genome Sequence of *Galleria mellonella* (Greater Wax Moth). *Genome Announcement* **6**, e01220-01217, doi:10.1128/genomeA.01220-17 (2018).
13 Ma, W. *et al.* A chromosome-level genome assembly reveals the genetic basis of cold tolerance in a notorious rice insect pest, *Chilo suppressalis*. *Molecular Ecology Resources* **20**, 268-282, doi:10.1111/1755-0998.13078 (2020).
14 Emms, D. M. & Kelly, S. OrthoFinder: solving fundamental biases in whole genome comparisons dramatically improves orthogroup inference accuracy. *Genome Biology* **16**, 1-14, doi:10.1186/s13059-015-0721-2 (2015).
15 Katoh, K. & Standley, D. M. MAFFT multiple sequence alignment software version 7: improvements in performance and usability. *Molecular Biology and Evolution* **30**, 772-780, doi:10.1093/molbev/mst010 (2013).
16 Price, M. N., Dehal, P. S. & Arkin, A. P. FastTree: computing large minimum evolution trees with profiles instead of a distance matrix. *Molecular Biology and Evolution* **26**, 1641-1650, doi:10.1093/molbev/msp077 (2009).
17 Sanderson, M. J. r8s: inferring absolute rates of molecular evolution and divergence times in the absence of a molecular clock. *Bioinformatics* **19**, 301-302, doi:10.1093/bioinformatics/19.2.301 (2003).
18 Wang, K. *et al.* De novo genome assembly of the white-spotted flower chafer (*Protaetia brevitarsis*). *GigaScience* **8**, 1-9, doi:10.1093/gigascience/giz019 (2019).
19 Bie, T. D., Cristianini, N., Demuth, J. P. & Hahn, M. W. CAFE: a computational tool for the study of gene family evolution. *Bioinformatics* **22**, 1269-1271, doi:10.1093/bioinformatics/btl097 (2006).
20 El-Gebali, S. *et al.* The Pfam protein families database in 2019. *Nucleic Acids Research* **47**, D427-D432 (2018).
21 Finn, R. D., Clements, J. & Eddy, S. R. HMMER web server: interactive sequence similarity searching. *Nucleic acids research* **39**, W29-W37, doi:10.1093/nar/gkr367 (2011).

22 Altschul, S. F., Gish, W., Miller, W., Myers, E. W. & Lipman, D. J. Basic local alignment search tool. *Journal of Molecular Biology* **215**, 403-410, doi:10.1016/S0022 -2836(05)80360 -2 (1990).
23 Vizueta, J., Sanchez-Gracia, A. & Rozas, J. bitacora: A comprehensive tool for the identification and annotation of gene families in genome assemblies. *Molecular Ecology Resoures* **20**, 1445-1452, doi:10.1111/1755-0998.13202 (2020).
24 Li, F. *et al.* Insect genomes: progress and challenges. *Insect Molecular Biology* **28**, 739-758, doi:10.1111/imb.12599 (2019).
25 van der Geest, L. P. S. & Evenhuis, H. H. *World Crop Pests 5: Tortricid pests their biology, natural enemies and control*. Vol. 5 (Elsevier, 1991).
26 Dritsou, V. *et al.* A draft genome sequence of an invasive mosquito: an Italian Aedes albopictus. *Pathogens and Global Health* **109**, 207-220, doi:10.1179/2047773215Y.0000000031 (2015).
27 Papanicolaou, A. *et al.* The whole genome sequence of the Mediterranean fruit fly, Ceratitis capitata (Wiedemann), reveals insights into the biology and adaptive evolution of a highly invasive pest species. *Genome Biology* **17**, 31, doi:10.1186/s13059-016-1049-2 (2016).
28 Xie, W. *et al.* Genome sequencing of the sweetpotato whitefly *Bemisia tabaci* MED/Q. *Gigascience* **6**, 1-7, doi:10.1093/gigascience/gix018 (2017).
29 Guo, S. K. *et al.* Chromosome-level assembly of the melon thrips genome yields insights into evolution of a sap-sucking lifestyle and pesticide resistance. *Molecular Ecology Resources*, 10.1111/1755-0998.13189, doi:10.1111/1755-0998.13189 (2020).
30 Jones, M. M., Robertson, J. L. & Weinzierl, R. A. Susceptibility of oriental fruit moth (Lepidoptera: Tortricidae) to two pyrethroids and a proposed diagnostic dose of esfenvalerate for field detection of resistance. *Journal of economic entomology* **104**, 1031-1037, doi:10.1603/ec10399 (2011).
31 Kanga, L. H., Pree, D. J., Van Lier, J. L. & Walker, G. M. Management of insecticide resistance in oriental fruit moth (Grapholita molesta; Lepidoptera: Tortricidae) populations from Ontario. *Pest management science* **59**, 921-927 (2003).
