# Supplementary material for: Population genomic signatures of the oriental fruit moth related to the Pleistocene climates
Source: Commun Biol. 2022 Feb 17;5:142. doi: 10.1038/s42003-022-03097-2 (PMC8854661; doi:10.1038/s42003-022-03097-2)
Supplement: Supplementary file 3 — Description of Additional Supplementary Files [file 42003_2022_3097_MOESM3_ESM.pdf]

Description of Additional Supplementary Files

File name: Supplementary Data 1
Description: Genes on the inverted regions of Grapholita molesta (Busck).

File name: Supplementary Data 2
Description: Annotation of outlier genes potentially involved in northward dispersal and endemicity of SCCD population.

File name: Supplementary Data 3
Description: KASP primers designed for 22 SNPs potentially under selection. Lines shaded in green indicated five primers revealed a stable amplification across the 12 populations.
