# Supplementary material for: Population genomic signatures of the oriental fruit moth related to the Pleistocene climates
Source: Commun Biol. 2022 Feb 17;5:142. doi: 10.1038/s42003-022-03097-2 (PMC8854661; doi:10.1038/s42003-022-03097-2)
Supplement: Supplementary file 7 — Reporting Summary [file 42003_2022_3097_MOESM7_ESM.pdf]

nature portfolio

Corresponding author(s): Shu-Jun Wei

Last updated by author(s): Dec 21, 2021

Reporting Summary

Nature Portfolio wishes to improve the reproducibility of the work that we publish. This form provides structure for consistency and transparency in reporting. For further information on Nature Portfolio policies, see our Editorial Policies and the Editorial Policy Checklist.

Statistics

For all statistical analyses, confirm that the following items are present in the figure legend, table legend, main text, or Methods section.

n/a	Confirmed	
☐	☒	The exact sample size (*n*) for each experimental group/condition, given as a discrete number and unit of measurement
☐	☒	A statement on whether measurements were taken from distinct samples or whether the same sample was measured repeatedly
☒	☐	The statistical test(s) used AND whether they are one- or two-sided<br>*Only common tests should be described solely by name; describe more complex techniques in the Methods section.*
☒	☐	A description of all covariates tested
☐	☒	A description of any assumptions or corrections, such as tests of normality and adjustment for multiple comparisons
☒	☐	A full description of the statistical parameters including central tendency (e.g. means) or other basic estimates (e.g. regression coefficient) AND variation (e.g. standard deviation) or associated estimates of uncertainty (e.g. confidence intervals)
☐	☒	For null hypothesis testing, the test statistic (e.g. *F*, *t*, *r*) with confidence intervals, effect sizes, degrees of freedom and *P* value noted<br>*Give P values as exact values whenever suitable.*
☐	☒	For Bayesian analysis, information on the choice of priors and Markov chain Monte Carlo settings
☒	☐	For hierarchical and complex designs, identification of the appropriate level for tests and full reporting of outcomes
☒	☐	Estimates of effect sizes (e.g. Cohen's *d*, Pearson's *r*), indicating how they were calculated

Our web collection on statistics for biologists contains articles on many of the points above.

Software and code

Policy information about availability of computer code

Data collection	No software tool is involved in data collection.
Data analysis	Genome assembly: CANU version v1.8, Purge Haplotigs, Pilon v1.22,3D de novo assembly (3D-DNA), Juicer v1.5<br>Genome annotation: MAKER v3.01.03, PASA v2.4.1,Augustus v3.2.3 , SNAP v2013-02-16, RepeatMasker v4.0.7, tRNAscan-SE, RNAmmer, Hisat v2.2.0, StringTie v2.1.2, Cufflinks v2.2.1, eggNOG-Mapper v1.0.3<br>Genome survey and assessment: GenomeScope v1.0, jellyfish v2.2.10, Universal Single-Copy Orthologs (BUSCO) v3.0.2, MCSCAN<br>Genotyping: BWA v0.7.17, SAMtools v1.9, Genome Analysis Toolkit (GATK) v3.5, R package vcfR, VCFtools v0.1.16, SNP_Primer_Pipeline2<br>Population genetic: VCFtools v0.1.16, PopLDdecay, KimTree v1.3, SMC++ v1.15.4, lostruct v.0.0.0.9, Bedtools v2.2.80, SnpEff v4.3, R package clusterProfiler

For manuscripts utilizing custom algorithms or software that are central to the research but not yet described in published literature, software must be made available to editors and reviewers. We strongly encourage code deposition in a community repository (e.g. GitHub). See the Nature Portfolio guidelines for submitting code & software for further information.

Data

Policy information about availability of data

All manuscripts must include a data availability statement. This statement should provide the following information, where applicable:

- Accession codes, unique identifiers, or web links for publicly available datasets
- A description of any restrictions on data availability
- For clinical datasets or third party data, please ensure that the statement adheres to our policy

The genome assembly has been deposited in the Genome repository (accession numbers: CP053120-CP053147) under NCBI BioProject PRJNA627114. The datasets used in data analysis are available in the Dryad repository (https://doi.org/10.5061/dryad.6wwpzgmzm).

Field-specific reporting

Please select the one below that is the best fit for your research. If you are not sure, read the appropriate sections before making your selection.

☒ Life sciences ☐ Behavioural & social sciences ☐ Ecological, evolutionary & environmental sciences

For a reference copy of the document with all sections, see nature.com/documents/nr-reporting-summary-flat.pdf

Life sciences study design

All studies must disclose on these points even when the disclosure is negative.

Sample size	263 individuals from 15 geographical populations of the oriental fruit moth in China.
Data exclusions	None.
Replication	No immediate replication is discussed.
Randomization	Randomization is not relevant to our study design.
Blinding	Blinding is not relevant to our study design.

Reporting for specific materials, systems and methods

We require information from authors about some types of materials, experimental systems and methods used in many studies. Here, indicate whether each material, system or method listed is relevant to your study. If you are not sure if a list item applies to your research, read the appropriate section before selecting a response.

Materials & experimental systems

n/a	Involved in the study	
☒	☐	Antibodies
☒	☐	Eukaryotic cell lines
☒	☐	Palaeontology and archaeology
☐	☒	Animals and other organisms
☒	☐	Human research participants
☒	☐	Clinical data
☒	☐	Dual use research of concern

Methods

n/a	Involved in the study	
☒	☐	ChIP-seq
☒	☐	Flow cytometry
☒	☐	MRI-based neuroimaging

Animals and other organisms

Policy information about studies involving animals; ARRIVE guidelines recommended for reporting animal research

Laboratory animals	A laboratory-reared strain of the oriental fruit moth was used in this study. This strain was derived from three male and female pairs collected from a orchard.
Wild animals	All larvae of the oriental fruit moth were collected from damaged fruits or tree shoots, preserved in absolute ethanol and stored at -80°C prior to DNA extraction.
Field-collected samples	A laboratory-reared strain of the oriental fruit moth was maintained on apples in laboratory conditions for ten generations.
Ethics oversight	Not applicable.

Note that full information on the approval of the study protocol must also be provided in the manuscript.
